# Dynamics of African swine fever virus shedding and excretion in domestic pigs infected by intramuscular inoculation and contact transmission

**DOI:** 10.1186/s13567-014-0093-8

**Published:** 2014-09-26

**Authors:** Claire Guinat, Ana Luisa Reis, Christopher L Netherton, Lynnette Goatley, Dirk U Pfeiffer, Linda Dixon

**Affiliations:** Pirbright Institute, Ash road, Woking, Surrey GU24 0NF UK; Veterinary Epidemiology, Economics and Public Health Group, Royal Veterinary College, University of London, Hatfield, AL9 7TA Hertfordshire UK

## Abstract

African swine fever virus (ASFV) is a highly virulent swine pathogen that has spread across Eastern Europe since 2007 and for which there is no effective vaccine or treatment available. The dynamics of shedding and excretion is not well known for this currently circulating ASFV strain. Therefore, susceptible pigs were exposed to pigs intramuscularly infected with the Georgia 2007/1 ASFV strain to measure those dynamics through within- and between-pen transmission scenarios. Blood, oral, nasal and rectal fluid samples were tested for the presence of ASFV by virus titration (VT) and quantitative real-time polymerase chain reaction (qPCR). Serum was tested for the presence of ASFV-specific antibodies. Both intramuscular inoculation and contact transmission resulted in development of acute disease in all pigs although the experiments indicated that the pathogenesis of the disease might be different, depending on the route of infection. Infectious ASFV was first isolated in blood among the inoculated pigs by day 3, and then chronologically among the direct and indirect contact pigs, by day 10 and 13, respectively. Close to the onset of clinical signs, higher ASFV titres were found in blood compared with nasal and rectal fluid samples among all pigs. No infectious ASFV was isolated in oral fluid samples although ASFV genome copies were detected. Only one animal developed antibodies starting after 12 days post-inoculation. The results provide quantitative data on shedding and excretion of the Georgia 2007/1 ASFV strain among domestic pigs and suggest a limited potential of this isolate to cause persistent infection.

## Introduction

African swine fever virus (ASFV) was introduced into Georgia in 2007 and continues to spread across Eastern European countries [1–4]. ASFV is a large enveloped DNA virus, the only member of the *Asfarviridae* family, genus *Asfivirus* [5], that naturally infects domestic and wild swine. African swine fever (ASF) is characterised by fever, haemorrhages and high mortality rates resulting in significant economic losses in affected areas [1,6]. There is no effective treatment or vaccine available so disease control is based on strict quarantine restrictions and stamping out measures [7]. The main routes reported for ASFV transmission are direct contact between infectious and susceptible domestic pigs and indirect contact through contaminated pork, people, vehicles, and fomites [1]. In Eastern Africa, ASFV is also transmitted by ticks but their epidemiological role in Eastern Europe has never been reported [1]. There is a risk that ASFV will spread further throughout Europe [1]. Therefore, it is important to obtain a better understanding of ASFV shedding and excretion within domestic pig farms so that transmission parameters can be appropriately estimated and then used to inform dynamics models of disease spread. These would allow the potential impact of various control policies to be assessed.

The incubation period, i.e. the time from infection to onset of clinical signs, the latent period, i.e. the time from infection to onset of infectiousness, and the infectious period, i.e. the time from onset of infectiousness to death or recovery, are important epidemiological parameters for describing the dynamics of shedding and excretion for infectious diseases [8]. They have not been quantified in experimental studies for the highly virulent Georgia 2007/1 ASFV strain. So far, there is no evidence for change in the virulence of this ASFV strain and infections continue to result in the acute form of the disease with no recovered or long-term carrier domestic pigs reported [1]. Incubation, latent and infectious periods are dependent on factors, such as the quantity of virus shedding and the route of virus excretion, which may in turn depend on the infectious dose and the virus strain [9]. Recent infection studies, with the Armenia ASFV strain, have focused on wild boar [10,11]. Following inoculation, the wild boar developed clinical signs after an incubation period from 3 to 4 days, virus shedding after a latent period from 2 to 6 days and all of them died between 7 and 9 days post-inoculation (dpi) [10,11]. ASFV-specific antibodies have been rarely reported in tested serum and organ samples from domestic pigs and wild boar from affected areas in Eastern Europe, supporting the view that acute disease results in death before the development of a detectable antibody response [1]. Other experimental infections have mainly studied the susceptibility of domestic pigs to infection with ASFV strains that circulate in Africa [12–16]. After inoculation with these strains, domestic pigs generally started to shed virus in blood after a latent period from 1 to 7 days. Some of them, infected with lower virulent ASFV strains, became persistently infectious for more than 70 dpi [12]. During the infectious period, they excreted higher ASFV titres through the oral route than for the nasal and rectal routes [12–16].

To our knowledge, no detailed information is available about the dynamics of virus shedding and excretion of the ASFV strain that is currently circulating among domestic pigs in Eastern Europe. We therefore investigated clinical signs, viremia and virus excretion patterns in domestic pigs which became infected by intramuscular inoculation and contact transmission with the Georgia 2007/1 ASFV strain in a controlled environment. The implications of these disease parameters for disease detection and transmission are discussed.

## Material and methods

### Animals and housing

Forty specific pathogen-free female Large White pigs (*Sus scrofa domesticus*) aged 7 weeks, coming from the same herd in the United Kingdom, were used. Animals were identified individually and randomly housed in four independent isolation rooms (width × length: 385–423 cm × 550 cm) within a containment Level 4 facility at the Pirbright Institute, Surrey, United Kingdom. Animals were fed twice a day by the animal caretakers and water was provided *ad libitum*. We calculated the minimum number of pairs of infectious/contact pigs providing high power (0.95) to detect a significant difference (0.05) in transmission between a control and treatment group. For this calculation, we considered the probability of infection *p* in each group defined as *p* = R0/(R0 + 2) [17] with R0, the basic reproduction number, i.e. the average number of newly infected cases caused by one infectious individual during its infectious period in a susceptible population [18]. Based on R0-control = 6.9 [19] and R0-treatment = 0.5, at least 4 pairs of infectious/contact pigs were required. Calculations were carried out using the R package “epi.studysize” [20]. Therefore, ten pigs were allocated to room A. Twelve pigs were allocated to rooms B and C, in which eight pigs were separated from the other four by an 80 cm high partition, so that between-pen transmission could be quantified. The sample size was slightly modified for room D as the number of pigs available and the isolation room size were limited. For these reasons, only six pigs were allocated to room D. All animal experiments were carried out under UK Home Office Licence number 70/7198 with the approval of the animal ethics committee at the Pirbright Institute and complied fully with the regulated procedures from the Animals (Scientific Procedures) Act 1986.

### Virus strains

The highly virulent Georgia 2007/1 ASFV strain was initially isolated from an infected pig originating from the Imereti Province in western Georgia in 2007 [21] and is genetically very close to ASFV strains that currently circulate in the Trans Caucasian countries and in the Russian Federation [22,23]. Virus was prepared from infected spleen tissue and the animals were inoculated with 1 mL of virus at 10^2^ 50% hemadsorbing doses (HAD_50_). The inoculum of 10^2^ HAD_50_ was chosen as this amount of virus was previously shown to efficiently induce infection by the intramuscular route [9].

### Experimental infection and transmission

Domestic pigs were inoculated intramuscularly, after a 5-day acclimatisation period. While the intramuscular route does not represent the natural infection, it appears to be the most reliable manner of challenge, allowing high incidence of infection, control of dose and timing of challenge [9]. The inocula were back-titrated to confirm the administered dose. Table 1 shows the number of inoculated and contact pigs per room. Five out of ten pigs were infected in room A, four out of twelve pigs were inoculated in room B, four out of twelve pigs were inoculated in room C and three out of six pigs were inoculated in room D. The remaining pigs served as within-pen contact pigs if they were in the same pen as the inoculated pigs or as between-pen contact pigs if they were in the adjacent pen.

**Table 1:** **Experimental infection and transmission results with the Georgia 2007/1 ASFV strain**

**Room**	**A**	**B**	**C**	**D**
No. inoculated pigs	5	4	4	3
No. within-pen contact pigs	5	4	4	3
No. between-pen contact pigs	0	4	4	0
No. naturally infected pigs	5	8	8	3
Samples*	B, NS, OS, RS, R			

*B: Blood, OS: Oral swab, NS: Nasal swab, RS: Rectal swab, R: Rope.

### Clinical and post-mortem examination

Pigs were examined daily for clinical signs. To assess the severity of the disease, a list of ten clinical signs has been defined and expressed quantitatively using a clinical score (CS) system [24]. Pigs with a rectal temperature over 40.5 °C for three consecutive days or showing three different clinical signs of disease were euthanized in accordance with the welfare regulations specified in the UK Home Office Licence under which animal experiments with ASFV are carried out at the Pirbright Institute. Post-mortem examination was performed for each pig and collected tissues (tonsil, spleen, kidney, lung, heart, lymph nodes) were examined for types of macroscopic lesions in accordance with the standardized pathological framework of ASFV infections [25].

### Sampling procedures

Individual pigs were removed from the room during sample collection in order to avoid contamination. Small pigs were restrained in dorsal recumbency and large pigs remained standing, restrained with a nasal snare. Between successive samplings, all materials necessary for blood sampling, clothing, footwear, gloves and floors were cleaned and disinfected. Blood and serum samples were collected every two days starting at day 3 post-inoculation until the end of the experiment. Oral, nasal and rectal samples were collected daily starting at day 2 post-inoculation using cotton swabs and soaked in 1 mL PBS. Negative control samples were collected at day 0, the day of inoculation. Oral swabs were taken between the cheeks and molar teeth. Fluids were collected from the cotton swabs after vortexing. Additional oral fluid was collected daily from a pressed rope hung in each room which could be chewed by the animals. All samples were stored at −80 °C until they were analysed by quantitative real-time polymerase chain reaction (qPCR) and virus titration (VT).

### Quantitative real-time polymerase chain reaction

Samples were analysed by qPCR to determine the quantity of ASFV genome copies, according to the procedure described in King et al. [26] with slight modifications. The DNA was extracted with the QIAamp® All Nucleic Acid Kit MDx Kit (Qiagen, UK) using an automated Qiagen Universal BioRobot (Qiagen, UK). After the DNA extraction procedure, the cartridge was processed for the qPCR. The target for amplification of the ASFV genome was the conserved p72 gene segment, using the following primers: 5′-CTG CTC ATG GTA TCA ATC TTA TCG A-3′ and 5′-GAT ACC ACA AGA TC(AG) GCC GT-3′. Analysis was performed using the MxPro software and the qPCR procedure included the following step: denaturation (95 °C), annealing (58 °C) and elongation (72 °C). The quantity of ASFV genome was calculated using the standard curve and expressed as genome copies per millilitre (/mL).

### Virus titration

Samples were analysed by VT to determine the quantity of infectious ASFV. Primary porcine bone marrow cells were incubated in 96-well plates at 37 °C in an atmosphere with 5% CO_2_. After 3 days, the medium was discarded and fresh medium (Eagle minimum essential medium (EMEM) with 10% of pig serum, 1:250 of HEPES and 2% of antibiotics solution (Penicillin, Streptomycin, Amphotericin, Kanamycin)) was added to the wells. Samples were added to the plates and titrated in triplicate using dilutions ranging from 10^-1^ to 10^-8^. After 3 days, the quantity of ASFV was determined by identification of characteristic rosette formation representing hemadsorption of erythrocytes around infected cells. ASFV titres were expressed as doses per millilitre (HAD_50_/mL) calculated using the Spearman-Karber method [27].

### Detection of ASFV-specific antibodies

Serum samples were tested for ASFV-specific antibodies with the Ingezim PPA Compac kit (Ingenasa, Madrid, Spain), according to the manufacturer’s instructions. This detects levels of antibodies against the VP72 (or VP73 capsid protein). Negative and positive cut-off values were calculated to interpret the results.

### Statistical analysis

For all clinical signs and viral patterns recorded, the mean ± standard deviation was calculated for the three groups, inoculated, within- and between-pen contact pigs, using data for individual pigs obtained daily. For the inoculated pigs, we calculated the average duration of the latent and the incubation period. Since data on the day of infection were not available for the contact pigs, the average duration between the exposure and the onset of infectiousness or clinical signs were calculated. For all animals, we calculated the average duration of the infectious period although all pigs were euthanized for animal welfare reasons. The average duration of each of the different time periods was compared between groups using a one-way ANOVA test. The onset of clinical signs and virus shedding were compared per group using a t-test. The type of post-mortem lesions were compared between groups using a one-way ANOVA test. The values of clinical scores and viral shedding were compared between groups in relation to time using a linear mixed effect model. All statistical analyses and plots were performed using the R statistical program [20]. The packages “epi.studysize”, “lme4”, “lmerTest”, “latticeExtra” and *p* < 0.05 were used to indicate statistical significance.

## Results

### Inoculation and transmission

All study pigs were susceptible to the Georgia 2007/1 ASFV strain resulting in infection of all animals as a result of intramuscular inoculation or contact transmission (Table 1). One inoculated pig survived until day 12 post-inoculation, but all other inoculated pigs were euthanized by day 9. All within- and between-pen contact pigs were euthanized by day 14 and day 18 post-exposure, respectively.

### Macroscopic lesions

**Figure 1:**
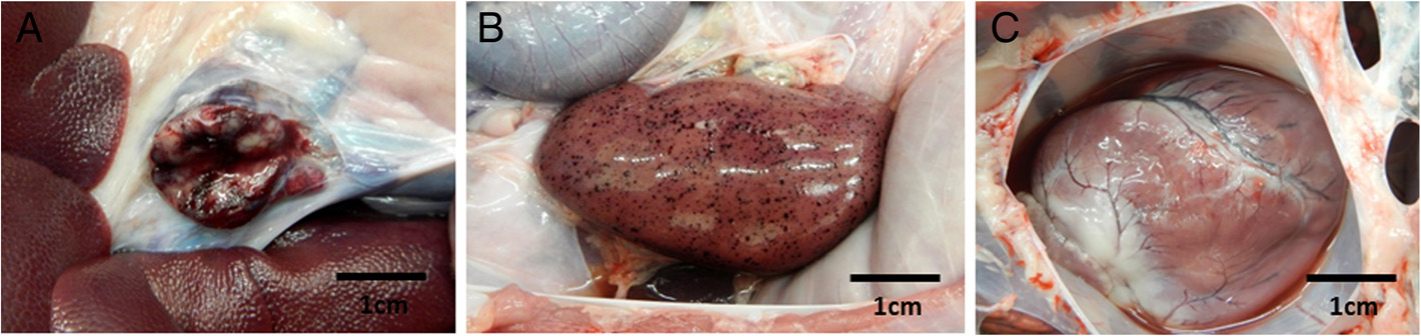
**Macroscopic lesions observed in organs of domestic pigs infected with the Georgia 2007/1 ASFV strain. A)** Enlarged and haemorrhagic hepatogastric lymph node from a between-pen contact pig terminated at 11 days post exposure. **B)** Multifocal cortical haemorrhages (petechiae) on kidney from an inoculated pig terminated at 8 days post-inoculation. **C)** Fluid in pericardial cavity (hydropericardium) from a within-pen contact pig terminated at 12 days post-exposure.

Figure 1 shows the most common macroscopic lesions observed for the three groups of pigs at post-mortem examination. There was no significant difference in the type of lesions among the three groups. We observed enlarged and haemorrhagic lymph nodes (submandibular, mesenteric, tracheo-bronchial and hepatogastric), enlarged and haemorrhagic spleen, multifocal cortical haemorrhages (petechiae) on lung, kidney and liver, and accumulation of fluid in the pericardial cavity (hydropericardium).

### Clinical signs and incubation period

**Figure 2:**
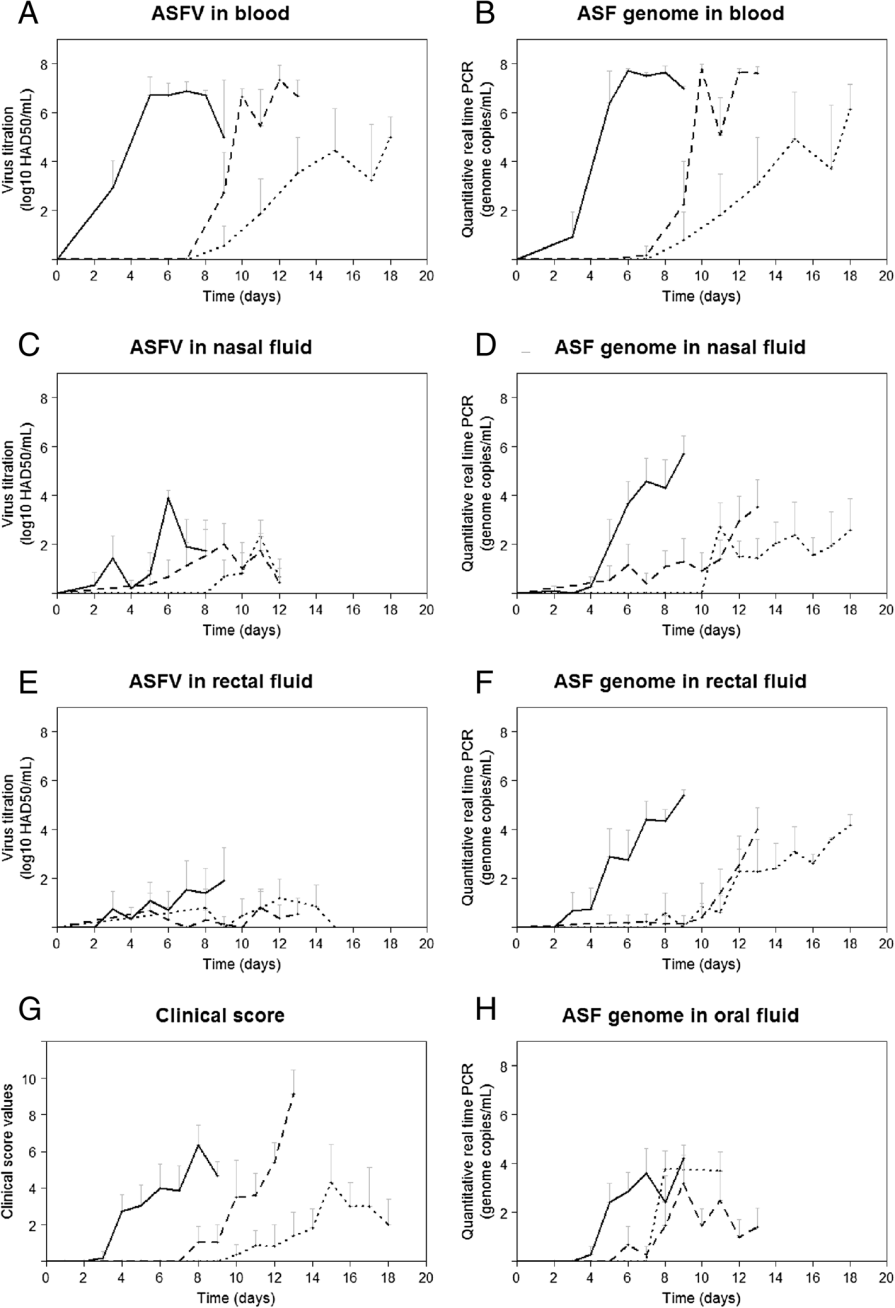
**Results of clinical signs, viremia and virus excretion patterns observed in domestic pigs infected intramuscularly (solid line type) with the Georgia 2007/1 ASFV strain, by direct contact (dashed line type) or by indirect contact (dotted line type). A)** ASFV titres in blood. **B)** ASF genome copies in blood. **C)** ASFV titres in nasal fluid. **D)** ASF genome copies in nasal fluid. **E)** ASFV titres in rectal fluid. **F)** ASF genome copies in rectal fluid. **G)** Clinical score. **H)** ASF genome copies in oral fluid. Means and standard deviations per time (days) are shown for all inoculated, within- and between-pen contact pigs.

Most pigs showed fever (higher than 40 °C for more than two consecutive days), loss of appetite, lethargy and dysentery. Table 2 shows the average duration of the incubation period for the inoculated pigs and the time to onset of clinical signs for the contact pigs. There was a significant difference in time to the appearance of clinical signs among the three groups. The inoculated pigs first started to show clinical signs at 4.4 ± 1.0 dpi, followed chronologically by the within- and the between-pen contact pigs, at 9.9 ± 1.6 and 12.7 ± 2.0 days post-exposure (dpe), respectively. The increase in clinical scores per day was significantly different between groups and tended to be higher for the inoculated pigs than for the within- and between-pen contact pigs, i.e. 0.5 ± 0.1/day and 0.3 ± 0.1/day respectively (Figure 2G).

**Table 2:** **Results of the average duration of latent and incubation period for the inoculated pigs and time to onset of infectiousness and clinical signs for the contact pigs**

	**Inoculated pigs**	**Within-pen contact pigs**	**Between-pen contact pigs**
**Samples**	Latent period*	**Time to onset of infectiousness†**	
Blood ‡	4.8 (± 1.3)	10.3 (± 1.6)	13.9 (± 3.0)
Blood §	3.6 (± 1.0)	10.4 (± 1.4)	13.1 (± 3.0)
Oral swab ‡	5.4 (± 1.3)	8.5 (± 1.5)	9.2 (± 1.5)
Nasal swab ‡	5.4 (± 1.4)	7.6 (± 2.6)	11.3 (± 0.5)
Rectal swab ‡	4.9 (± 1.4)	9.3 (± 2.9)	11.0 (± 1.6)
	Incubation period*	Time to onset of clinical signs†	
Clinical score >3	4.4 (± 1.0)	9.9 (± 1.6)	12.7 (± 2.0)

*Average number of days post inoculation (± standard deviation).

†Average number of days post exposure (± standard deviation).

‡Results by quantitative real-time polymerase chain reaction.

§Results by virus titration.

### Latent period and time to onset of infectiousness

Table 2 shows the average duration of the latent period for the inoculated pigs and the time to onset of infectiousness for the contact pigs, by ASFV genome or infectious ASFV detection in blood, oral, nasal and rectal samples. There was a significant difference in time to the appearance of infectious ASFV in blood among the three groups. The appearance of ASFV genome in blood among the three groups was not significantly different from infectious ASFV. In the inoculated pigs, infectious ASFV was first isolated in the blood at 3.6 ± 1.0 dpi, and generally two days later in the swabs. In the within-pen contact pigs, infectious ASFV was isolated in the blood at 10.4 ± 1.4 dpe, although ASFV genome could be detected in swabs a few days before. In the between-pen contact pigs, infectious ASFV was isolated in the blood at 13.1 ± 3.0 dpe, although ASFV genome could be also detected earlier in swabs. Considering the standard deviation, values for the time to onset of viremia tended to spread over a larger range within the contact pigs than within the inoculated pigs. The time to onset of infectious ASFV in blood and clinical signs were not significantly different among the contact pigs while the difference was significant for the inoculated pigs. Infectious ASFV was occasionally isolated in nasal and rectal swabs among the three groups while oral swabs remained negative (Figure 2C and 2E). Oral fluid collected from the ropes indicated the presence of ASFV genome from day 5 in the room A and from day 13 in the room C but no infectious ASFV was recovered from these samples. No ASFV-specific antibodies were detected except from one inoculated pig at 12 dpi.

### Detection of ASFV genome and infectious ASFV

Figure 2 shows the average values of infectious ASFV and ASFV genome detected in blood, oral, nasal and rectal swabs and the clinical signs for the three groups of pigs. Highest ASFV titres were mainly found in blood ranging from 10^6^ to 10^8^ HAD_50_/mL (Figure 2A) and small amounts were occasionally detected in nasal and rectal swabs ranging from 10^2^ to 10^4^ HAD_50_/mL (Figure 2C and 2E). The increase of the ASFV titres in blood per day was significantly different between groups and tended to be higher for the inoculated pigs than for the within- and between-pen contact pigs, 0.5 ± 0.1 log_10_ HAD_50_/mL/day compared to 0.3 ± 0.1 log_10_ HAD_50_/mL/day, respectively. Considering the standard deviation, larger range of ASFV titres in blood were observed within the contact pigs than for the inoculated pigs (Figure 2A). Infectious ASFV was occasionally isolated in nasal and rectal fluid samples although high ASFV genome copies values were detected (Figure 2D and 2F). No infectious ASFV was isolated in oral fluid samples although ASFV genome copies were detected (Figure 2H).

### Infectious period

Table 3 shows the average duration of the infectious period for each of the three groups of pigs by ASFV genome and infectious ASFV detection in blood, oral, nasal and rectal samples. There was a significant difference in the duration of the infectious period among the three groups. ASFV genome and infectious ASFV were detected within blood from the inoculated pigs for a significantly longer time compared to the contact pigs. In addition, ASFV genome and infectious ASFV were detected in oral, nasal and rectal swabs among the inoculated pigs for a significantly shorter time compared to the contact pigs.

**Table 3:** **Results of the average duration of infectious period**

	**Inoculated pigs**	**Within-pen contact pigs**	**Between-pen contact pigs**
**Samples**	Infectious period*		
Blood †	3.5 (± 1.3)	2.4 (± 1.2)	2.3 (± 0.9)
Blood ‡	4.7 (± 1.4)	2.4 (± 0.7)	3.0 (± 1.2)
Oral swab †	3.2 (± 1.2)	4.8 (± 1.5)	7.7 (± 3.3)
Nasal swab †	2.9 (± 1.3)	5.0 (± 2.3)	5.4 (± 1.4)
Rectal swab †	3.4 (± 1.2)	3.8 (± 2.3)	5.7 (± 1.7)

*Average number of days (± standard deviation).

†Results by quantitative real-time polymerase chain reaction.

‡Results by virus titration.

## Discussion

The objective of the current study was to provide quantitative information on the dynamics of shedding and excretion of the Georgia 2007/1 ASFV strain among domestic pigs. Results confirmed that pigs are highly susceptible to direct and indirect infection with the currently circulating ASFV strain in Eastern Europe resulting in development of the acute disease form. While one inoculated pig survived until day 12, all others were euthanized by day 9. All within- and between-pen contact pigs were euthanized by day 14 and 18, respectively. This work suggests that the Georgia 2007/1 ASFV strain has limited potential in controlled environment to cause persistent ASFV infection or result in ASFV carriers in a pig population as was also suggested for the wild boar [11]. The infection caused moderate and non-specific clinical signs and lesions among all pigs [25]. In recent publications, similar clinical signs and macroscopic lesions were observed in wild boar and domestic pigs during infection experiments using a similar ASFV strain [10,11] and during ASF outbreaks in domestic pigs in the affected geographical areas [1,6]. The inoculated pigs generally started to show clinical symptoms after 3 days post-inoculation, meaning that significant delay could be possible between infection and disease reporting within pig farms. These clinical signs and lesions can be misdiagnosed with other haemorrhagic diseases such as classical swine fever and porcine dermatitis and nephropathy syndrome resulting not only in potential underreporting but also in major delay for ASFV diagnosis [28,29]. Therefore, ASFV could spread rapidly through transport of animals between farms, markets and slaughter houses, without farmers suspecting and reporting ASFV infection.

This study also shows that the disease transmission is likely to occur by contact with blood, as was suggested for wild boar [11]. The average duration of the latent period/onset of infectiousness was 3.6 ± 1.0, 10.4 ± 1.4 and 13.1 ± 3.0 days for the inoculated, within- and between-pen contact pigs respectively, according to the blood samples. The between-pen contact pigs became viremic significantly later than the within-pen contact pigs, indicating that specific farm contact infrastructures, such as fenced premises, could delay the disease transmission. The three groups showed high ASFV titres in blood compared to swabs reaching from 10^6^ to 10^8^ HAD_50_/mL. Recent studies on domestic pig behaviour reported that common social interactions, such as feeding or mating, generally cause skin injuries and if this induces any bleeding then this appears to give rise to more bites and licking [30]. Therefore, blood would easily contaminate the environment, especially in a within-pen transmission scenario. The amount of infectious ASFV detected in the blood was comparable to the number of ASFV genome copies detected by qPCR, highlighting the utility of this assay in disease control. However, for a few blood samples from the inoculated pigs, the latent period was estimated to be shorter, when tested by virus titration compared to qPCR. The reasons for these discrepant results are unknown, however out of the 222 blood samples tested for the presence of ASFV genome using the OIE qPCR assay [26], eight samples were misdiagnosed, remaining consistent with the sensitivity of this assay [31–34]. There was no significant difference in time to the onset of viremia and clinical signs in the contact pigs, indicating that natural transmission mostly occurs at the same time as the ASF disease signs appear. This last observation emphasizes that early detection based on clinical signs would not be an efficient approach for in-farm control for this ASFV strain in contrast to other infectious diseases where such measures led to the successful eradication [35].

Moreover, these experiments show that the disease transmission is also possible through oral, nasal and rectal fluids. These excretions could be easily spread in the environment by the natural explorative behaviour of domestic pigs resulting in a high chance for ASFV to transmit, especially in a between-pen transmission scenario [30]. The onset of infectiousness was detected through the nasal or oral route for the within- and between-pen contact pigs before these animals showed viremia. However, infectious ASFV was excreted at lower levels via these routes compared to blood, reaching from 10^2^ to 10^4^ HAD_50_/mL. Besides, ASFV isolation in nasal and rectal swabs rarely produced positive results. Inconsistent with reports from other studies [10–12], no ASFV was isolated in oral swabs or from ropes samples. This suggests that either ASFV survival may be reduced through the oral route by potential presence of inhibitors in saliva or that ASFV in samples was rapidly inactivated and unsuitable for detection by virus titration, although false positive results due to contamination in the qPCR assay cannot be excluded [36]. ASFV genome was generally detected in oral and nasal samples from the contact pigs before they developed viremia. This could be explained by the fact that ASFV appears to first replicate in monocytes and macrophage cells from lymph nodes close to the initial site of infection [37]. Hence, ASFV would first replicate in the oropharyngeal region when pigs are infected by contact transmission resulting in excretion of infectious virus through the oral and nasal routes prior to systemic dissemination. This also indicates that detection of ASFV genome in oronasal swabs would be a relevant tool for early diagnosis of infected pigs as it has been suggested for CSFV [38].

Several factors from the experiments demonstrate that the pathogenesis of the disease might be different, depending on the route of infection. For the inoculated pigs, the first detection of ASFV genome was in blood while it was in oral and nasal swabs for the contact pigs, suggesting earlier systemic spread with the intramuscular inoculation. Moreover, the increase of the ASFV titres in blood per day was significantly different between the three groups and tended to be higher for the inoculated pigs, suggesting a faster disease progression with the intramuscular inoculation. Similar results were reported for the increase of the clinical scores although the type of clinical signs did not vary according to the route of infection. Also smaller standard deviations in infection and shedding patterns were observed among the inoculated pigs, suggesting reduced natural heterogeneity with the intramuscular inoculation. Finally, there was a significant difference in the duration of the infectious period among the three groups although all the animals were euthanized before their natural death. This variation in pathogenesis may be due to different primary sites of replication for the intramuscular and natural infection routes, affecting the dynamics of ASFV shedding and excretion [9,37]. Consequently, data from the inoculated pigs could not be considered as representative of the natural course of ASFV infection as data from the contact pigs. These observations stress that further effort is needed to obtain more accurate information on the course of ASFV infection. Although probably less effective than the intramuscular route, other inoculation routes could be considered [9] but also additional transmission experiments with the use of secondary contact infection [39] or modelling approaches [8]. All pigs were euthanized before their death caused by the disease for animal welfare reasons, resulting in possible bias for the infectious period duration estimations. However, domestic pigs were previously reported to generally be infectious for similar time intervals, i.e. from 3 to 6 days [11]. Finally, serology results showed that ASFV-specific antibodies were detected in only one inoculated pig at 12 dpi. Previous studies have described the important role of antibody detection in the control of the disease for low virulence ASFV strains [40]. In the affected areas in Eastern Europe, animals have only been reported to develop the acute form of the disease so far and mostly died at a very early stage of the disease, before the appearance of ASFV specific antibodies [1]. Therefore, our results may imply that serology does not provide a reliable method for disease surveillance in the context of the current circulating ASFV strain unless the virulence of ASFV strains reduces such that longer term or persistent infections could occur.

Results should be considered carefully as many factors, including virus shedding, exposure time, virus survival, sample size and others experimental conditions may influence the outcome of the transmission studies. As an example, variability was observed in ASFV shedding or time to onset of infectiousness within the contact pigs. This could be explained by individual susceptibility to infection or specific type of contacts between infectious and susceptible pigs that would determine transmission efficiency. However, this experimental study proved to have the main advantages of requiring a limited number of animals, allowing frequent animals sampling and environmental factors control compared to field studies. In all experiments, ASFV transmission occurred, suggesting that the disease could spread easily among domestic pigs within pig farms. However, field observations indicated that during ASF outbreaks, the pig mortality rate could be low and that despite the high virulence of the Georgia 2007/1 ASFV strain, healthy susceptible pigs were reported within infected herds [41]. This also implies that disease transmission may occur at different rates probably as a result of host characteristics or animal husbandry [18,42,43]. In conclusion, these results provide the first quantitative information to be used for estimating transmission parameters for ASFV and thus developing future dynamic transmission models of within-herd ASFV spread. This will have potential applications for the development and implementation of transmission control policies.
